# Supplementary material for: Respiratory Outcomes of Insulin Use in Patients with COPD: A Nationwide Population-Based Cohort Study
Source: Pharmaceuticals (Basel). 2023 Apr 24;16(5):643. doi: 10.3390/ph16050643 (PMC10222134; doi:10.3390/ph16050643)
Supplement: Supplementary file 1 [file pharmaceuticals-16-00643-s001.zip › pharmaceuticals-2324935-supplementary.pdf]

Table S1. Diseases and related ICD-9-CM, ICD-10-CM codes.

Disease	ICD-9-CM Codes	ICD-10-CM Codes
Type 2 diabetes	250.xx, except 250.1x	E11
Chronic obstructive pulmonary disease	491, 492, or 496	J41, J42, J44, J43, or J44.9
Type 1 diabetes	250.1x	E10
Dialysis	V56.0, V56.8, V45.1	Z49.31, Z49.32, Z99.2
Hepatic failure	570, 572.2, 572.4, 572.8	K72.00, K72.01, K72.10, K72.11, K72.90, K76.2, K72.90, K72.91, K76.7, K76.81
Obesity	278.02, 783.1, V85.2, 278.00, 649.1, V77.8, V85.3, 278.01, 649.2, V45.86, V85.4	R63.5, E66.09, E66.1, E66.8, E66.9, Z13.89, E66.01, E66.2
Smoking status	305.1, 649.0, V15.82	F17.200, F17.201, F17.210, F17.220, F17.221, F17.290, F17.291, Z87.891
Alcohol-related disorders	291, 303, 305.0, 571.0-571.3, V11.3, V79.1	F10, K70.40, K70.41, K70.9
Hypertension	401–405, A26	I10, I11, I12, I13, I15, N26
Dyslipidemia	272	E71.30, E71.31, E71.32, E71.39, E75.21, E75.22, E75.23, E75.24, E75.25, E75.29, E75.3, E75.4, E75.5, E75.6, E77, E78.0, E78.1, E78.2, E78.3, E78.4, E78.5, E78.6, E78.70, E78.71, E78.72, E78.79, E78.8, E78.9
Coronary artery disease	410-414	I20, I21, I22, I24, I25.1, I25.2, I25.3, I25.4, I25.5, I25.6, I25.7, I25.81, I25.82, I25.83, I25.84, I25.89, I25.9
Atrial fibrillation	427	I45.0, I45.1, I45.2, I45.3, I45.4, I45.5, I45.6
Peripheral arterial disease	440.0, 440.20, 440.21, 440.22, 440.23, 440.24, 440.3, 440.4, 443.9, 443.81, 443.89	I70.2, I70.92, I75.0, I73.9
Chronic kidney disease	250.4x, 403.xx, 404.xx, 585.xx, 586.xx, 581.8x, 791.0x, 593.9x, V42.0x, V45.1x, V56.0x, V56.8x, 39.27, 39.42, 39.43, 39.49, 39.50, 39.53, 39.93, 39.94, or 39.95	E10.2, E10.65, E11.2, E11.65, E13.2, I12, I13, N03, N08, E10.21, E11.21, N05, N06, N07, N14, N15.0, N15.8, N15.9, N16, N17.1, N17.2, N18, N19, Z94.0, Z49.31, Z49.32, Z99.2, Z94.0
Liver cirrhosis	571.5, 571.2, 571.6	K70.2, K70.30, K70.31, K74.0, K74.1, K74.2, K74.60, K74.69, K74.3, K74.4, K74.5

Lung cancer	162.0, 162.2, 162.3, 162.4, 162.5, 162.8, 162.9	C34.00-C34.92
Non-invasive positive pressure ventilation	93.90, 93.91	Z99.81
Invasive mechanical ventilation	96.7	Z99.1
Bacterial pneumonia	480-486	J12-18
Hypoglycemia	251.0x, 251.1x, or 251.2x	E16.0, E16.1, E16.2
